# Enlarged Area of Mesencephalic Iron Deposits in Adults Who Stutter

**DOI:** 10.3389/fnhum.2021.639269

**Published:** 2021-02-11

**Authors:** Jan Liman, Alexander Wolff von Gudenberg, Mathias Baehr, Walter Paulus, Nicole E. Neef, Martin Sommer

**Affiliations:** ^1^Department of Neurology, University Medical Center Göttingen, Göttingen, Germany; ^2^Institut der Kasseler Stottertherapie (KST), Bad Emstal, Germany; ^3^Department of Clinical Neurophysiology, University Medical Center Göttingen, Göttingen, Germany

**Keywords:** stuttering, mesencephalic iron, transcranial ultrasound, dopamine, finger tapping, walking

## Abstract

**Purpose:**

Childhood onset speech fluency disorder (stuttering) is possibly related to dopaminergic dysfunction. Mesencephalic hyperechogenicity (ME) detected by transcranial ultrasound (TCS) might be seen as an indirect marker of dopaminergic dysfunction. We here determined whether adults who stutter since childhood (AWS) show ME.

**Methods:**

We performed TCS in ten AWS and ten matched adults who never stuttered. We also assessed motor performance in finger tapping and in the 25 Foot Walking test.

**Results:**

Compared to controls, AWS showed enlarged ME on either side. Finger tapping was slower in AWS. Walking cadence, i.e., the ratio of number of steps by time, tended to be higher in AWS than in control participants.

**Discussion:**

The results demonstrate a motor deficit in AWS linked to dopaminergic dysfunction and extending beyond speech. Since iron deposits evolve in childhood and shrink thereafter, ME might serve as an easily quantifiable biomarker helping to predict the risk of persistency in children who stutter.

## Introduction

Stuttering is a speech fluency disorder with involuntary repetitions of syllables and sounds, prolongations of sounds and blockages (American Psychiatric Association, 2013). Attempts to stop or avoid a stuttering event result in accompanying symptoms such as excessive muscular tension, co-movements of muscles not normally involved in speech (Mulligan et al., 2001), or verbal and situational avoidance. In its most prevalent form, stuttering occurs in about 5% of all children and persists in about 1% of adults, mostly males (Yairi and Ambrose, 2013). It is then termed persistent developmental stuttering (PDS) and can negatively impact career and personal development (McAllister et al., 2012).

The cause of stuttering is unknown. Studies suggest a disconnection of speech-related brain regions based on structural imaging findings, and an over activity of dopaminergic metabolism in people who stutter (Alm, 2004). The latter hypothesis is based on (1) clinical, (2) pharmacological and (3) neurophysiological as well as (4) imaging evidence.

(1) Clinically there are similarities with basal ganglia disorders (Alm, 2004), namely (a) the intermittent nature of speech dysfluencies, more frequently correlated with stress and excitement and impairment of motor control (Yoshie et al., 2009). There is (b) a task specificity for speech without clinically discernible involvement of other features of the articulatory organs such as swallowing or chewing. This is reminiscent of task-specific dystonias (Kiziltan and Akalin, 1996). In addition, (c) stuttering shares features with tic disorders, in particular with the Tourette’s syndrome. Males are predominantly affected in either of the two disorders (McNaught and Mink, 2011), and premonitory feelings of imminent dysfunctional states of the motor system occur in both disorders (Brandt et al., 2016; Cholin et al., 2016).
(2) Pharmacologically, a number of studies have shown an improvement in stuttering severity through intake of dopamine-antagonist neuroleptics (Maguire et al., 2020). On the other hand, L-dopa was reported to impair speech fluency in an individual afflicted with developmental stuttering and parkinson’s disease (PD) (Anderson et al., 1999). Syllable repetitions are frequent in PD patients, even though assessment may be complicated by concomitant dysarthria (Hertrich et al., 1994). In addition, deep brain stimulation of either suthalamic nucleus or globus pallidus internus may worsen preexisting childhood onset stuttering, or induce the occurrence of stuttering (Picillo et al., 2017).
(3) Neurophysiologically, the balance between inhibitory and excitatory motor intracortical interneurons is tilted in basal ganglia disorders such as Parkinson’s disease or dystonia, with less active intracortical inhibitory circuits (Ridding et al., 1995a,b). Adults who stutter since childhood (AWS) show a dysfunction mostly of the intracortical facilitatory circuitry (Neef et al., 2011). Interestingly, neuroleptics, at least in healthy individuals, increase intracortical facilitation (Paulus et al., 2008).
(4) Several fMRI studies report an altered involvement of the substantia nigra (SN) in speech and motor tasks and a correlation between SN activity and stuttering severity (Giraud et al., 2008; Watkins et al., 2008; Metzger et al., 2018). AWS showed an increased activity in the left globus pallidus and the left lateral thalamus in stuttered as compared to fluent reading (Fox et al., 1996).

To explore traits of dopaminergic dysfunction in AWS further, we here employed transcranial ultrasound (TCS) of the midbrain. TCS is useful for brain parenchyma assessment of iron deposits in the substantia nigra (Behnke et al., 2010). We here hypothesized that size and symmetry of mesencephalic iron deposits may be abnormal in AWS as compared to a fluent speaking control population. We specifically targeted the SN area as the best established target structure accessible to TCS and linked to dopaminergic function. Given the dynamic modulation of iron and neuromelanin in the early lifetime (Iova et al., 2004; Xing et al., 2018), we were unsure which direction of change (if any) to expect. Knowledge from neurodegenerative disorders cannot easily be extrapolated to neurodevelopmental disorders.

## Materials and Methods

### Participants and Methods

We obtained written informed consent from all participants, and the protocol was approved by the University Medical Center Göttingen Ethics Committee.

Participants would have been excluded in case they had other neurological diseases, stuttering other than childhood onset speech fluency disorder, drug and/or alcohol dependence, severe hearing deficit impairing normal communication, or inappropriate concomitant medication: iron as a tablet/capsule or as an infusion, or if they had participated in the study team. We recruited 11 AWS and 12 control participants. One AWS was excluded because of an insufficient temporal insonication window on either side, and two control participants could not be rescheduled for the ultrasound study. Hence, ten participants per group entered the final analysis. Participants were paid 10 EUR per hour.

### Speech Assessment

We quantified speech fluency using the Stuttering Severity Index (SSI-3). The SSI-3 provides a quantitative offline analysis of speech fluency (Riley, 1994). For this purpose, 1000 syllables were analyzed from speech samples of reading and spontaneous speech production, respectively, obtained within a standardized interview. The result is the percentage of stuttered syllables. In addition, the duration of the event and the quality of any physical reactions that may occur are estimated for all stuttering subjects, resulting in an overall SSI score. This assessment was done by a qualified speech language pathologist. Stuttering severity in the AWS group ranged from 20 to 47, with a median of 32.5 and an interquartile range of 12.3. One of the ten AWS was categorized as mild, three as moderate, tree as severe, and three as very severe.

### Ultrasound Procedure

The ultrasound examinations took place in a resting, relaxed state. We used a standard, commercially available ultrasonic device (Siemens X3000 Professional, Erlangen, Germany) with a phased array ultrasonic probe (1.5–3.5 MHz, Dynamic Range 45–50 db, penetration depth 14–16 cm). The probe was placed on the temporal bone (temporal bone window) analogous to the TCS used for studying intracranial vessels (axial incision). First, the level of the 3rd ventricle was searched. In this level, the third ventricle, the lateral ventricles and the cranial nerve roots are assessed. The ventricles were measured in diameter, and the ganglia assessed for their echogenicity (hypo, iso, or hyperechoic with respect to the adjacent brain parenchyma).

Subsequently, the mid-brain plane was searched as the plane in which the substantia nigra is located. We measured it planimetrically and calculated its area. Furthermore, a semiquantitative assessment was made with respect to the adjacent mesencephalic structure (analogous to the root ganglia).

### Motor Behavior

Even though the impairment in stuttering is clinically task-specific and confined to speech fluency, detailed observation of hand motor tasks did in fact reveal subtle impairments, too (Webster and Ryan, 1991; Zelaznik et al., 1994). This points to a more generalized motor problem, and motivated us to study basic motor behavior of hand function and walking in our sample, with the intention to correlate this with ultrasound findings on the one hand and severity of stuttering on the other hand. Therefore, we assessed handedness using the Oldfield handedness questionnaire (Oldfield, 1971). In addition, we quantified basic motor behavior through the maximum number of finger taps within 20 s, assessed by using a cell counter activated by thumb adduction movements, using two runs at either side (Sommer et al., 2002). We also assessed speed and number of steps in a Timed 25 Foot Walk performed twice (Motl et al., 2017). In addition, a board-certified neurologist assessed the unified parkinson’s disease rating scale (UPDRS) part III in all participants (Fahn et al., 1987).

### Data Analysis

We analyzed the SN area using a repeated-measures analysis of variance (ANOVA) with “side” (left, right) as within-subjects-factor and “group” (control, AWS) as between-subjects factor. We analyzed finger tapping using a repeated-measures ANOVA with “side” (left, right) and “run” (1, 2) as within-subjects-factor and “group” (control, AWS) as between-subjects factor. For analysis of walking cadence (Kidziński et al., 2020), we entered the ratio of the number of steps and the walking time, averaged across two runs, into a factorial ANOVAs with “group” (control, AWS) as between-subjects factor. StatView 5.0 (SAS, Cary, NC, United States) was used for initial data assessment, and SPSS 26.0 (IBM Inc., Armonk, NY, United States) for analysis, tests of sphericity and calculation of effect sizes. Mauchly’s test of sphericity confirmed that repeated-measures ANOVAs were adequate.

Using Pearson’s correlation coefficient in the group of AWS, we correlated SN area on either side, as well as the average SN area of both sides, with stuttering severity as quantified by the SSI total score, with the number of steps, with the individual walking cadence (ratio of number of steps by time) and with the number of finger taps on either side, as well as with the average number of taps across both sides.

## Results

Table 1 lists epidemiological details of the participants.

**TABLE 1:** Demographic data of participants.

Measures	AWS	Controls	Significance
Participants, *n*	10 (7M, 3F)	10 (7M, 3F)	–
Age (years), mean	27.27 (SD = 4.82)	26.35 (SD = 2.13)	*p* = .663 (n.s.)
Handedness, mean	60.00 (SD = 79.44)	31.45 (SD = 80.43)	*p* = .112 (n.s.)
Height (cm)	177.40 (SD = 7.71)	182.00 (SD = 13.93)	*p* = .540 (n.s.)
Percentage of syllables stuttered, mean	18.20 (SD = 16.11)	0.04 (SD = 0.10)	*P* = .021 (sig.)
SSI-3 Mean Overall Score	32.80 (SD = 7.97)	3.60 (SD = 3.06)	*P* < .0001 (sig.)
Age of onset (years)	5.15 (SD = 3.47)	–	–
UPDRS III score (points)	.70 (SD = 1.16)	1.50 (SD = 1.43)	*P* < .162 (n.s.)

*We used an unpaired, two-tailed *t*-test for age and Mann-Whitney *U*-Tests for all other measures.*

**FIGURE 1:**
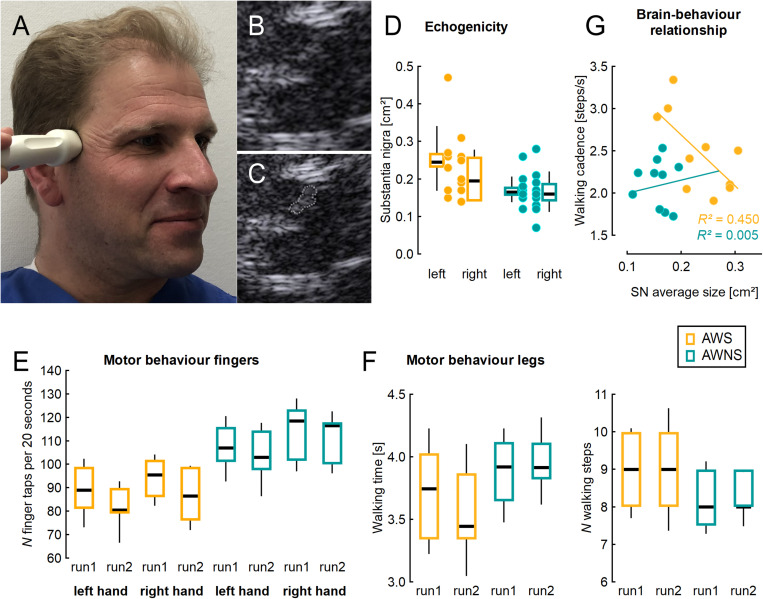
**(A)** Illustration of ultrasound probe placement, **(B)** typical example from mesencephalic ultrasound, **(C)** same figure as in **B** with the shape of the substantia nigra measured planimetrically. **(D)** Area of the substantia nigra measured planimetrically by TCS on either side, individual values (dots) and adjacent box plots. **(E)** Number of finger taps per 20 s, two runs for each side studied. **(F)** Time and number of steps needed to perform a Timed 25 Foot Walk task, two runs studied. **(G)** Correlation of walking cadence with the average SN area of both sides, in the group of AWS. AWS, adults who stutter, AWNS, adults who do not stutter. Box plots illustrate median, 25th and 75th percentile; the whiskers indicate one standard deviation above and below the mean.

Figure 1 shows a typical example. SN was larger in AWS than in controls [effect of group, *F*(1,17) = 13.77 *p* = 0.0017; η2 = 0.45; see Figure 1B], with no effect of side or interaction of side by group.

Finger tapping was slower in AWS than in controls [effect of group, *F*(1,18) = 13.13; *p* = 0.002; η^2^ = 0.42; see Figure 1C]. In both groups, it worsened across runs [effect of run, *F*(1,18) = 25.15; *p* < 0.0001; η^2^ = 0.58], and was slower on the left side [effect of side, *F*(1,18) = 11.27; *p* = 0.004; η^2^ = 0.39], without any interaction.

There was a trend for walking cadence to be higher in AWS (2.48 SD = 0.48) than in AWNS (2.12 SD = 0.28; unpaired, two-tailed *t*-test, *p* = 0.054; η^2^ = 0.19).

Correlation analyses were unrevealing except for the walking cadence which showed a good correlation with the average SN area (Pearson’s correlation coefficient, *r* = −0.67; *r*^2^ = 0.45) (see Figure 1E) among AWS, but not among ANS (*r* = 0.07; *r*^2^ = 0.005). In AWS, this correlation between SN area and walking cadence was carried more by the right SN (Pearson’s correlation coefficient, *r* = −0.63; *r*^2^ = 0.40) than by the left SN (Pearson’s correlation coefficient, *r* = −0.33; *r*^2^ = 0.11).

## Discussion

We found an enlarged SN area in AWS. To our knowledge, mesencephalic TCS has never been explored in stuttering.

The size and echogenicity of the SN as detected on ultrasound is largely determined by iron accumulation, as confirmed by human post-mortem studies (Berg et al., 2002). Since the 1990s, an enlarged mesencephalic area of iron accumulation has been linked to dopaminergic deficit disorders (Becker et al., 1995). It is a trait marker of Lewy body pathology rather than state marker of disease course, since it was present in the absence of clinically apparent Parkinson’s disease (Berg et al., 1999, 2002) and unchanged after five years of disease duration despite clinical worsening (Berg et al., 2005). In children, hyperechogenicity is high and decreases until the age of 10 years (Iova et al., 2004).

Excessive mesencephalic and midbrain iron accumulation is the pathological hallmark of Pantothenate kinase-associated neurodegeneration (PKAN). In these patients, hyperechogenicity of the SN was found compared to healthy controls (Liman et al., 2012). Similarly, in patients with cervical and upper limb dystonia, TCS displayed increased lenticular nucleus echogenicity pronounced contralateral to the clinically affected side (Becker et al., 1997). An increased copper and manganese content in the lenticular nucleus compared to controls could be ruled out via magnetic reconance imaging (MRI) (Becker et al., 1999), in line with other studies proofing good co-localization of iron deposits in the midbrain in T2^∗^ weighted images compared to ultrasound (Ahmadi et al., 2020). In the large and ever-growing literature studying MRI in children and adults who stutter, these specific sequences –at least to our knowledge– have not been investigated.

The pathophysiological role of these mesencephalic iron accumulations is not fully understood. There is a link to enzymes of the dopaminergic pathway requiring iron for proper functioning (Zucca et al., 2017). Iron accumulation in mesencephalic nuclei is supposed to trigger oxidative stress, and thereby neurodegeneration (Berg et al., 2006).

On the other hand, neuromelanin is a scavenger protein removing excess substances, including metal ions (Xing et al., 2018). On autopsy, it is the neuromelanin that gives that area a black appearance, hence the name “substantia nigra” (Dickson, 2012). Neuromelanin content in the midbrain increases during the first years of life and decreases later on. Hence, the time course is inverse of what has been described for iron (Xing et al., 2018). It is conceivable that as the scavenger function declines, iron excessively accumulates.

There is a gender imbalance in that females are less likely to accumulate iron in the midbrain (Mahlknecht et al., 2013; Yu et al., 2018), and less likely to develop Parkinson’s disease (Haaxma et al., 2007). Of note, females are also less likely to show persistent stuttering than males (Yairi and Ambrose, 2013). Whether this is coincidental or causally linked is unknown.

Indeed, a minority of PKAN patients present with speech fluency disorders, which can precede the onset of other motor symptoms (Zhang et al., 2019; Natteru and Huang, 2020). By contrast, hypoechogenicity of the midbrain has been reported in restless-legs syndrome and taken as indicating reduced iron deposition (Schmidauer et al., 2005).

The direction of the observed group difference was difficult to predict. Our finding is not easy to integrate into a simple concept, in which a neurodegenerative disorder with a dopaminergic deficit such as Parkinson’s disease has a higher echogenicity, whereas a non-degenerative disorder benefiting also from dopaminergic therapy such as restless-legs syndrome has a lower echogenicity. Stuttering individuals improve to some extent by dopamine receptor antagonists (Maguire et al., 2020) and also show ME. In general, neurodevelopmental as compared to a neurodegenerative disorder may behave differently.

The basic motor behavior that we studied revealed slowed manual performance in AWS, consistent with the literature (Webster and Ryan, 1991; Zelaznik et al., 1994). We are not aware of an earlier study on walking in AWS. Our data suggest that AWS employ a strategy different from controls to perform the 25 foot walking test, using many fast and small steps rather than few longer steps. Given the dopaminergic influence on step length (Palmisano et al., 2020), this unexpected finding may be worth further study. Body height was similar in both groups, and is therefore unlikely to have caused the group difference. Taken together, the data from motor behavior indicates subtle motor deficits beyond the speech domain in AWS.

In stuttering, an excess of striatal dopamine has been postulated, based on one FDOPA PET study (Wu et al., 1995) and the clinical effect of dopamine receptor antagonists (Maguire et al., 2020). As higher dopamine levels are associated with faster movement, such a hyperdopaminergic state is difficult to reconcile with the speech and non-speech motor deficits observed in AWS. One way to integrate these observations is that of hyperkinesia associated with high dopamine levels. Indeed, excess movements can worsen movement performance, as exemplified by falls due to hyperkinesia in PD. One could speculate about an inverted center-surround concept (Mink, 1996), where the surround is disinhibited, thereby inducing excess movements that impair performance (Vreeswijk et al., 2018). One mechanism of excess movements to impair performance is an increased agonist- antagonist co-activation, as observed in musicians (Yoshie et al., 2009) or healthy volunteers (Yoshie et al., 2016) under stressful conditions. Of course, all these speculations need to be substantiated experimentally.

One long-standing theory of stuttering postulated an abnormal cerebral lateralization (Travis, 1978). This was based on early observations of handedness, which were, however, not consistently reproduced later on [see review chapter in Bloodstein and Ratner (2008)]. Theoretically, one could have expected ME to be unilateral in stuttering, but this was not the case. Indeed, an asymmetry of ME was found only recently in a large sample of more than hundred individuals at risk of developing PD, with a stronger ME contralateral to the dominant hand (Iranzo et al., 2020). We assume that our sample size is much too limited to detect subtle side differences.

## Conclusion

We conclude that there is a moderate ME in AWS, likely related to excess of iron deposits. A next clinically relevant step will be to assess mesencephalic iron accumulation in children who stutter, to see whether this can serve as early predictor of recovery or persistency of stuttering.

## Data Availability Statement

The raw data supporting the conclusions of this article will be made available by the authors, without undue reservation.

## Ethics Statement

The studies involving human participants were reviewed and approved by the University Medical Center Göttingen Ethics Committee. The patients/participants provided their written informed consent to participate in this study. Written informed consent was obtained from the individual(s) for the publication of any potentially identifiable images or data included in this article.

## Author Contributions

JL recorded the data, initiated the data analysis, and helped to write the manuscript. AW was active in participant recruitment and data recording. NN contributed to study design and discussed the results. WP and MB provided funding, commented on the analysis, and interpretation of the data. MS designed the experiments, assembled the setup, contributed to the statistical analysis of the data, and wrote the first draft. All authors discussed the results and commented on the manuscript.

## Conflict of Interest

MS serves as chairman of the German Stuttering Association (Bundesvereinigung Stottern & Selbsthilfe, e.V.), and also reports personal fees and grants from Deutsche Forschungsgemeinschaft, Primate Cognition (Leibniz-WissenschaftsCampus), Scientific Organizations (EFCN, UCL, DGKN, and IVS) and pharmaceutical companies (Novartis, GlaxoSmithKline, UCB, Medtronic), all outside the submitted work. AW is founder and head of the Institute of the Kassel Stuttering Therapy (KST), Reports contracts: with German medical insurance companies for the KST therapy programs, receives royalties for the software “flunatic” which the clients use in the therapy-programs of the KST, and reports a grant from Hessian Ministry of social affairs and health. WP reports personal fees and grants from Precisis AG, Bel Company, and Intellectual Property Rights-Patent on transcranial deep brain stimulation, all outside the submitted work. The remaining authors declare that the research was conducted in the absence of any commercial or financial relationships that could be construed as a potential conflict of interest.
